# Severe Parathyroid Hormone (PTH)-Independent Hypercalcemia Revealing Precursor B-cell Acute Lymphoblastic Leukemia in a Two-Year-Old Male: A Case Report

**DOI:** 10.7759/cureus.111191

**Published:** 2026-06-20

**Authors:** Sara Zbair, Fatima Zahra Yakine, Kamilia Chbani, Bouchra Slaoui

**Affiliations:** 1 Pediatric Endocrinology Unit, 2nd Pediatric Department, Abderrahim Harouchi Mother and Child Hospital, Ibn Rochd University Hospital, Casablanca, MAR; 2 Faculty of Medicine and Pharmacy, Hassan II University of Casablanca, Casablanca, MAR; 3 Pediatric Radiology Department, Abderrahim Harouchi Mother and Child Hospital, Ibn Rochd University Hospital, Casablanca, MAR; 4 Pediatric Pneumology and Allergology Unit, 2nd Pediatric Department, Abderrahim Harouchi Mother and Child Hospital, Ibn Rochd University Hospital, Casablanca, MAR

**Keywords:** acute lymphoblastic leukemia (all), case report, malignant hypercalcemia, osteolytic bone lesions, pediatric hypercalcemia, precursor b-cell lymphoblastic leukemia, pth-independent hypercalcemia

## Abstract

Hypercalcemia is an uncommon metabolic disorder in children and a rare complication of pediatric malignancies. Acute lymphoblastic leukemia (ALL) presenting with severe hypercalcemia and osteolytic bone lesions is rare in pediatric patients. We report the case of a previously healthy two-year-old male who presented with a one-month history of progressive lower limb weakness, fatigue, and altered responsiveness. Guillain-Barré syndrome was initially suspected but became less likely following normal electromyography (EMG) and spinal MRI findings. Initial investigations revealed severe hypercalcemia (212 mg/L; 5.3 mmol/L), hypophosphatemia, hyperuricemia, elevated lactate dehydrogenase (LDH) levels, and mildly impaired renal function. A shortened QT interval on ECG prompted pediatric ICU (PICU) admission, where the patient received hyperhydration and bisphosphonates, resulting in partial biochemical improvement. Endocrine evaluation showed suppressed parathyroid hormone (PTH) (4 pg/mL), normal 25-OH vitamin D (23 ng/mL), and normal thyroid function tests, supporting a PTH-independent etiology of hypercalcemia.

Persistent hypercalcemia combined with microcytic anemia, elevated uric acid, and LDH levels raised suspicion of an underlying malignancy. Skeletal radiographs demonstrated multiple bilateral osteolytic lesions involving the femurs, tibias, and fibulas. Bone marrow aspiration revealed 27% blasts, and flow cytometry demonstrated a CD34-positive and CD45-dim blast population consistent with precursor B-cell ALL. Serum calcium normalized progressively before chemotherapy initiation after initial aggressive hyperhydration followed by repeated zoledronic acid administration, and the patient was subsequently transferred to the oncology department for treatment according to the ALL IC-BFM 2009 protocol. Clinical and neurological improvement followed progressively.

This report highlights the diagnostic challenge of malignant hypercalcemia in children, particularly in the absence of circulating blasts or organomegaly. Severe PTH-independent hypercalcemia associated with osteolytic lesions should raise suspicion for ALL, even in very young children. Skeletal radiography provided the pivotal diagnostic clue after a one-month delay in diagnosis, ultimately leading to bone marrow evaluation and diagnosis.

## Introduction

Acute lymphoblastic leukemia (ALL) is the most common childhood malignancy, accounting for approximately one-third of all pediatric cancers and 75% of childhood leukemia cases [1]. Its classic presentation includes fever, pallor, bone pain, lymphadenopathy, and hepatosplenomegaly. However, ALL may occasionally manifest through atypical metabolic disturbances, particularly hypercalcemia, which can dominate the clinical picture and significantly delay diagnosis, especially in very young children.

Hypercalcemia is defined as a total serum calcium exceeding 105 mg/L (2.6 mmol/L) and is classified as severe above 140 mg/L (3.5 mmol/L). Values above 145 mg/L (3.6 mmol/L) are generally considered a hypercalcemic crisis, a life-threatening metabolic emergency associated with neuromuscular, cardiac, and renal complications [2]. In childhood, hypercalcemia is uncommon and encompasses a broad spectrum of etiologies, including nutritional, metabolic, genetic, endocrine, granulomatous, and neoplastic causes [3]. Malignancy-associated hypercalcemia is particularly rare in children, with a reported incidence of 0.4% to 1.3% among pediatric cancer patients [4]. Initial management relies on aggressive intravenous hydration and, in severe cases, bisphosphonate therapy, which remains the most effective pharmacological option for sustained reduction of serum calcium, particularly in malignancy-associated hypercalcemia [3].

Among pediatric hematological malignancies, ALL is the most commonly implicated cause of hypercalcemia at diagnosis. In a large retrospective series of over 6,000 pediatric cancer patients, approximately 40% of hypercalcemia cases were associated with leukemia [5]. Affected children frequently present with normal peripheral blood counts, absent circulating blasts, and no palpable organomegaly, features that substantially complicate early diagnosis [6]. Several mechanisms have been proposed to explain hypercalcemia in ALL. Parathyroid hormone-related peptide (PTHrP)-mediated hypercalcemia has been reported in some cases. It may contribute through stimulation of osteoclast-mediated bone resorption via the RANK/RANKL/OPG axis and enhanced renal calcium reabsorption [7]. Alternative PTHrP-independent pathways involving pro-inflammatory cytokines and other osteoclast-activating factors have also been described [8].

We report the case of a two-year-old male in whom severe PTH-independent hypercalcemia revealed precursor B-cell ALL. This case is particularly noteworthy because of the unusual combination of very young age, neuromuscular symptoms initially suggestive of Guillain-Barré syndrome, absence of circulating blasts and organomegaly, diffuse osteolytic lesions, and severe PTH-independent hypercalcemia associated with ECG abnormalities. Together, these atypical features contributed to diagnostic delay and highlight an important diagnostic pitfall that clinicians across multiple specialties should consider when evaluating persistent hypercalcemia in children.

This article was previously presented as a poster at the Sixth Arab Society of Pediatric Endocrinology and Diabetes (ASPED) Conference, held on September 26-27, 2025, in Dubai, United Arab Emirates.

## Case presentation

A previously healthy two-year-old male (two years and four months) was admitted to the Pediatric Endocrinology Unit of A. Harouchi Mother and Child Hospital, CHU Ibn Rochd, Casablanca, Morocco, following a one-month history of progressive symptoms and multiple prior evaluations. His past medical and family history were unremarkable, with no history of excessive calcium intake, thiazide use, prolonged immobilization, or familial hypercalcemia. Vitamin D supplementation had been administered as two intramuscular doses of cholecalciferol (100,000 IU each), given at birth and at six months of age, in accordance with the standard national prophylactic protocol. No additional doses were reported, and the last administration had occurred approximately 22 months before admission.

Initial presentation and emergency evaluation

Approximately one month before admission to our unit, the child had developed progressive bilateral lower limb weakness, profound fatigue, and altered responsiveness. He was initially evaluated in the pediatric emergency department, where Guillain-Barré syndrome was the leading diagnostic hypothesis. Electromyography (EMG) showed no abnormalities, making this diagnosis less likely, while spinal and brain MRI findings were unremarkable. Metabolic investigations subsequently revealed severe hypercalcemia, with corrected serum calcium at 212 mg/L (5.3 mmol/L), which redirected the diagnostic approach.

Pediatric intensive care unit (PICU) admission

Severe hypercalcemia associated with QT interval shortening on ECG warranted immediate PICU admission. The patient received aggressive intravenous hyperhydration and bisphosphonate therapy under continuous cardiac monitoring for four days, resulting in partial improvement of serum calcium levels before transfer to our unit. Exact QT/QTc measurements were not available retrospectively.

Evaluation in the pediatric endocrinology unit

On admission, the patient was hemodynamically stable with appropriate anthropometry for age. Clinical examination revealed mucocutaneous pallor, diffuse hypotonia, and reduced motor response in the lower limbs. No peripheral lymphadenopathy or organomegaly was detected.

A systematic etiological workup was conducted to identify the underlying cause of hypercalcemia. PTH was suppressed at 4 pg/mL (reference range: 15-65 pg/mL), supporting a PTH-independent mechanism and arguing against primary hyperparathyroidism. Repeat calcium-phosphorus assessment confirmed persistent hypercalcemia associated with hypophosphatemia. Serum albumin was 36 g/L, and calcium values reported in the manuscript correspond to corrected total serum calcium. The 25-OH vitamin D level was normal at 23 ng/mL, making vitamin D intoxication highly unlikely in this clinical context. Alkaline phosphatase was within the normal range, which did not support a primary metabolic bone disorder. Thyroid function tests (thyroid-stimulating hormone (TSH) 2.85 mIU/L, free T4 14 ng/L) were normal, arguing against thyrotoxicosis. Tumor markers, including alpha-fetoprotein (7.5 ng/mL) and beta-HCG (0.38 mIU/mL), were within normal limits, making hepatoblastoma and germ cell tumors unlikely. Procalcitonin was low at 0.14 ng/mL, and clinical assessment did not support bacterial sepsis.

Urinary investigations revealed hypercalciuria (urinary calcium 6.58 mmol/L, calcium-to-creatinine ratio 0.7), with preserved natriuresis (urinary sodium 45 mEq/L), consistent with hypercalciuria in the context of severe hypercalcemia. Renal function showed mild impairment, with creatinine at 5.5 mg/L and an estimated glomerular filtration rate of 67.6 mL/min/1.73 m², calculated using the bedside Schwartz formula. Renal ultrasound showed no nephrocalcinosis or structural abnormality. Serum electrolytes showed sodium at 135 mmol/L and potassium at 3.5 mmol/L, both within the normal range. Serum magnesium and 1,25-OH vitamin D levels were not available at admission.

Complete blood count revealed microcytic hypochromic anemia (hemoglobin (Hb) 7.2 g/dL) with a low-normal reticulocyte count (56,000/µL), consistent with a central aregenerative pattern. Ferritin and C-reactive protein (CRP) were elevated at 112 ng/mL and 17 mg/L, respectively. White blood cell count and platelet count were within normal ranges, with no circulating blasts identified. Elevated lactate dehydrogenase (LDH) (460 IU/L) and uric acid (118 mg/L) raised concerns for increased cell turnover and an underlying malignancy. Cervical and abdominal ultrasonography showed no significant abnormalities on initial assessment. All biological parameters and their evolution are summarized in Table 1.

**Table 1:** Evolution of key biological parameters over time Days 1-3 correspond to PICU stay. Day 4 corresponds to admission to the Pediatric Endocrinology Unit. Calcium values correspond to corrected total serum calcium. Values are presented as mg/L with mmol/L equivalents. Ionized calcium, serum magnesium, and 1,25-OH vitamin D levels were not available at admission Ca²⁺: serum calcium; PTH: parathyroid hormone; LDH: lactate dehydrogenase; Uric acid: serum uric acid; CRP: C-reactive protein; PCT: procalcitonin; AFP: alpha-fetoprotein; eGFR: estimated glomerular filtration rate; ALT: alanine aminotransferase; AST: aspartate aminotransferase; GGT: gamma-glutamyl transferase; WBC: white blood cell; PICU: pediatric intensive care unit

Parameter (Ref. range)	Unit	Day 1	Day 2	Day 3	Day 4	Day 5	Day 6	Day 7	Day 11	Day 16	Day 24
Calcium/phosphate/PTH											
Corrected serum Ca²⁺ (90–110)	mg/L	212	207	112	103	92	89	82	94	96	106
Corrected serum Ca²⁺	mmol/L	5.30	5.18	2.80	2.58	2.30	2.23	2.05	2.35	2.40	2.65
PTH (15–65)	pg/mL	4	—	—	7	—	26	—	—	—	16
Phosphorus (25–50)	mg/L	—	—	—	19	—	12	18	—	50	65
25-OH vitamin D (20–80)	ng/mL	—	—	—	23	—	—	—	—	—	—
Albumin (38–54)	g/L	36	—	—	—	—	—	—	—	—	—
Inflammatory and tumor markers											
LDH (<250)	IU/L	460	—	—	450	—	—	—	324	387	432
Uric acid (<70)	mg/L	118	—	—	118	—	—	28	12	23	21
CRP (<5)	mg/L	—	—	—	17	—	—	—	—	—	—
PCT (<0.5)	ng/mL	—	—	—	0.14	—	—	—	—	—	—
Ferritin (10–60)	ng/mL	—	—	—	112	—	—	—	—	—	—
AFP (<10)	ng/mL	—	—	—	7.5	—	—	—	—	—	—
Beta-HCG (<5)	mIU/mL	—	—	—	0.38	—	—	—	—	—	—
Renal function and electrolytes											
Creatinine (2.0–5.0)	mg/L	5.5	—	—	—	4.4	—	3.6	—	—	3.6
Urea (0.10–0.45)	g/L	—	—	—	—	—	—	—	—	—	—
eGFR (Schwartz) (≥90)	ml/min/1.73 m²	67.6	—	—	—	—	—	—	—	—	—
Sodium (136–145)	mmol/L	135	—	—	133	136	140	140	140	134	136
Potassium (3.4–4.7)	mmol/L	3.5	—	—	—	—	—	—	—	—	—
ALT/AST	IU/L	10/29	—	—	—	—	—	—	—	—	—
GGT (<45)	IU/L	14	—	—	—	—	—	—	—	—	—
Urinary Investigations											
Urinary Ca²⁺	mmol/L	6.58	—	—	—	—	—	—	—	—	—
Ca/Cr ratio (<0.21)	mg/mg	0.7	—	—	—	—	—	—	—	—	—
Urinary Na⁺	mEq/L	45	—	—	—	—	—	—	—	—	—
Urinary phosphorus	mEq/L	25	—	—	—	—	—	—	—	—	—
Complete blood count											
Hemoglobin (11.5–14.5)	g/dL	—	—	—	7.2	—	—	—	8.2	—	10
WBC (5–13 ×10³)	/µL	—	—	—	12,090	—	—	—	11,120	—	17,270
Neutrophils	/µL	—	—	—	5,390	—	—	—	3,860	—	4,920
Lymphocytes	/µL	—	—	—	5,970	—	—	—	6,320	—	10,170
Platelets (150–400 ×10³)	/µL	—	—	—	224,000	—	—	—	436,000	—	668,000
Zoledronic acid administration (0.03 mg/kg/dose IV)											
Dose 1 (PICU)	—	Yes	—	—	—	—	—	—	—	—	—
Dose 2 (PICU)	—	—	Yes	—	—	—	—	—	—	—	—
Dose 3 (endocrinology)	—	—	—	—	Yes	—	—	—	—	—	—
Dose 4 (endocrinology)	—	—	—	—	—	—	Yes	—	—	—	—

Pivotal imaging findings

**Figure 1:**
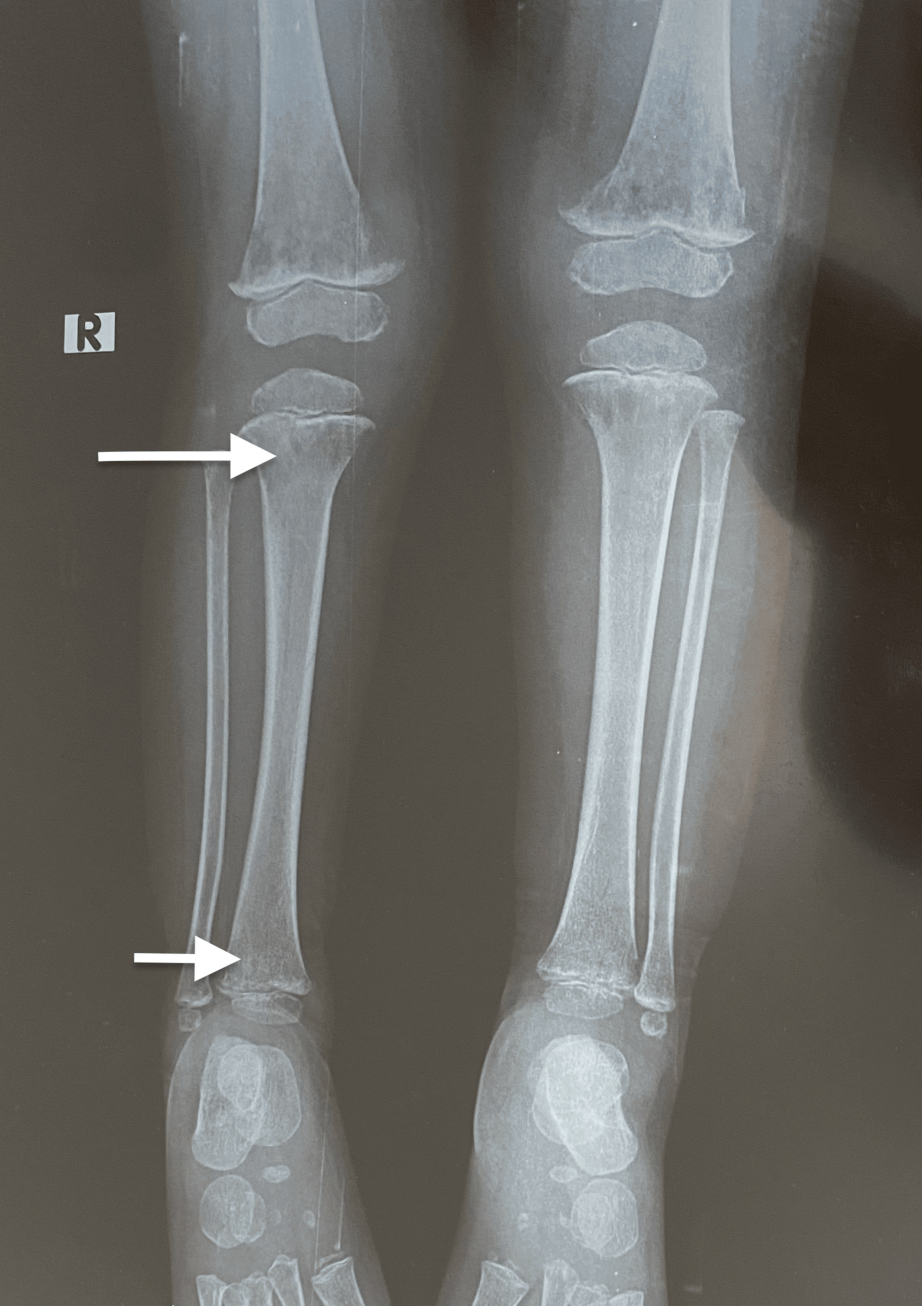
Plain radiograph of the lower limbs Diffuse bone rarefaction, bilateral osteolytic lesions of the tibias and fibulas, periosteal reaction, and metaphyseal radiolucent bands (“leukemic lines”) at the knee and ankle regions (arrows), consistent with leukemic bone infiltration. No pathological fracture is identified

Skeletal radiographs of the lower limbs provided the pivotal diagnostic clue in this case. Plain radiographs demonstrated bilateral osteolytic lesions of the tibias and fibulas, with characteristic metaphyseal radiolucent bands (“leukemic lines”) at the knee and ankle regions (Figure 1), without evidence of pathological fractures. These findings, in combination with suppressed PTH levels, elevated LDH and uric acid levels, and central aregenerative anemia, raised a strong suspicion of an underlying hematological malignancy.

A whole-body CT scan confirmed and extended the skeletal involvement. Cervical imaging identified bilateral cervical lymphadenopathies measuring up to 12 mm on the right and 10 mm on the left (level IIa). Thoracic imaging demonstrated ground-glass opacities with micronodular peribronchial infiltrates in the right lower lobe, without mediastinal lymphadenopathy or pleural effusion. Abdominal imaging revealed homogeneous hepatomegaly and mild right nephromegaly. Skeletal reconstruction showed diffuse osteolytic lesions involving both the axial and appendicular skeleton, associated with focal cortical disruption and multilevel vertebral compression fractures at T8, T12, and L5, as well as grade III collapse at T4 without posterior wall retropulsion.

Hematological diagnosis

Bone marrow aspiration revealed 27% blasts, confirming acute leukemia. Flow cytometry performed on bone marrow aspirate demonstrated a CD45-dim blast population (56.51%) expressing B-cell lineage markers, including CD19 (23.97%), CD22 (7.42%), CD10 (76.23%), intracytoplasmic CD79a (88.36%), CD20 (12.98%), and cytoplasmic immunoglobulin (24.85%), along with immaturity markers including CD34 (21.14%), HLA-DR (92.56%), intracytoplasmic TdT (42.70%), and CD38 (57.54%). Myeloid markers, including MPO, CD117, CD33, and CD13, and T-cell markers, including CD3 and CD7, were negative or only weakly expressed. The immunophenotypic profile was consistent with common B-cell precursor ALL (CD10-positive). Conventional cytogenetic analysis revealed a normal male karyotype (46, XY) without high-risk translocations, including t(9;22)/BCR-ABL1, t(4;11)/MLL-AF4, and t(17;19)/E2A-HLF.

Treatment and outcome

**Figure 2:**
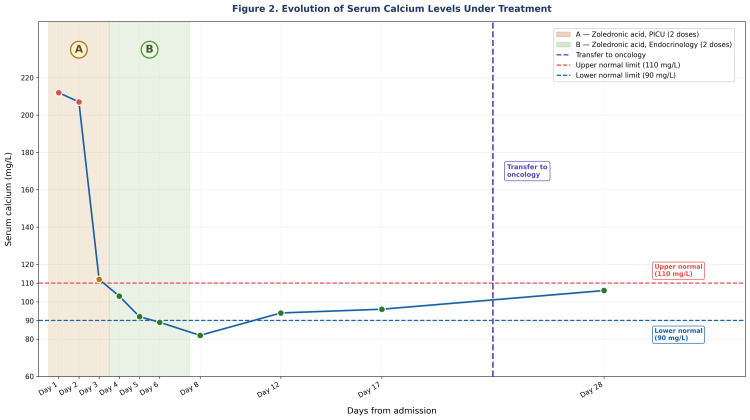
Evolution of serum calcium levels under treatment Serum calcium levels during hospitalization and follow-up. Orange zone (A): Two doses of zoledronic acid were administered during PICU admission with intravenous hyperhydration. Green zone (B): Two additional doses were administered in the Pediatric Endocrinology Unit. The dashed vertical line indicates transfer to the pediatric oncology department. Serum calcium normalized before chemotherapy initiation PICU: pediatric intensive care unit

Following diagnosis, the patient first received aggressive intravenous hyperhydration at 3 L/m²/day, initially using isotonic saline (0.9% NaCl) followed by a glucose-based solution, to promote urinary calcium excretion. He subsequently received four doses of intravenous zoledronic acid (0.03 mg/kg/dose). Serum calcium progressively normalized before chemotherapy initiation, as illustrated in Figure 2. PTH levels gradually recovered (4 → 7 → 26 pg/mL) alongside phosphorus normalization, supporting reversible PTH suppression after correction of severe hypercalcemia. The patient was subsequently referred to the pediatric oncology department for initiation of chemotherapy according to the ALL IC-BFM 2009 protocol. Neurological symptoms progressively resolved, and follow-up imaging demonstrated partial regression of bone lesions. No major complications occurred during induction therapy.

## Discussion

This case illustrates a rare and diagnostically challenging presentation of precursor B-cell ALL in a two-year-old toddler, in whom severe PTH-independent hypercalcemia was the dominant presenting feature. The stepwise diagnostic course across three clinical settings reflects the complexity of atypical ALL presentations and highlights several important learning points. The association between ALL and hypercalcemia at diagnosis remains uncommon, estimated at 0.4% to 1.3% of pediatric cancer patients [4,5]. As summarized in Table 2, most published cases involve older children, typically in the first or second decade of life, with a pre-B-cell immunophenotype, low or normal white blood cell counts, and absent circulating blasts [6,9]. Our case adds to this literature by illustrating this presentation in a very young child with severe hypercalcemia, diffuse osteolytic lesions, neuromuscular symptoms initially raising suspicion for Guillain-Barré syndrome, and absence of circulating blasts. Published pediatric ALL reports presenting with hypercalcemia and/or osteolytic lesions are summarized in Table 2.

**Table 2:** Summary of published pediatric ALL reports presenting with hypercalcemia and/or osteolytic lesions Ca²⁺ values represent total or corrected serum calcium, as reported in the original publications ALL: acute lymphoblastic leukemia; Ca²⁺: serum calcium; PTHrP: parathyroid hormone-related protein; CR: complete remission; mo: months; yrs: years; chemo: chemotherapy; BMT: bone marrow transplantation; SCT: stem cell transplantation; "—": not reported

Study	Patient age	Sex	Peak Ca²⁺	Osteolytic lesions	Circulating blasts	PTHrP measured	Treatment of hypercalcemia	Outcome
Dhivyasree et al. (2018) [2]	4 yrs	F	3.32 mmol/L	Yes (multiple long bones)	No	Not reported	Hydration + furosemide + pamidronate + calcitonin	Ca normalized after chemotherapy
Bonilla Gonzalez et al. (2022) [3]	12 yrs	M	4.71 mmol/L	No	No	Not reported	Hydration + furosemide + zoledronic acid, 0.05 mg/kg	Ca normalized 72 h after zoledronic acid; good response
Lin et al. (2021) [5]	35 mo	M	3.48 mmol/L	No	No	Not reported	Hydration + chemotherapy	Ca normalized by day 18; normal karyotype reported
Trehan et al. (2009) [6]	Various	—	Variable (review)	Variable	Often absent	Elevated in some	Hydration ± bisphosphonate ± chemotherapy	Variable (overview)
Shimonodan et al. (2005) [9]	14 yrs	F	3.65–5.20 mmol/L	Yes (multiple)	No	Elevated (PTHrP+)	Hydration + chemotherapy + BMT	CR then relapse; died of infection
Bahoush and Miri-Aliabad (2014) [10]	Child	—	Severe (>14 mg/dL)	Yes	No	Not reported	Hydration + bisphosphonate	Ca normalized; chemotherapy initiated
Park et al. (2016) [11]	15 yrs	M	3.55 mmol/L	Yes	Rare	Not reported	Hydration + zoledronic acid (single dose)	Ca normalized; good response to chemotherapy
Lokadasan et al. (2015) [12]	15 yrs	M	4.30 mmol/L	Yes (disseminated)	Occasional	Not reported	Hydration + bisphosphonate + chemotherapy	Ca normalized; referred for SCT
Bechir et al. (2017) [13]	3 yrs	M	Severe (exact value not reported)	Yes (skull, extremities, pelvis)	No	Not reported	Hydration + diuretics + corticosteroids + chemo	Ca normalized after chemo; relapsed; died of septic shock
Present case	2 yrs 4 mo	M	21.2 mg/dL 5.30 mmol/L	Yes (bilateral lower limbs + axial skeleton)	No	Not measured (unavailable)	Hydration + zoledronic acid 0.03 mg/kg/dose	Ca normalized before chemotherapy

A distinctive feature of this case was the shortened QT interval documented on ECG during PICU admission. Hypercalcemia shortens the QT interval by accelerating ventricular repolarization through abbreviation of the action potential plateau phase, which may increase the risk of ventricular arrhythmias [3]. This ECG abnormality justified ICU-level monitoring and illustrates that severe hypercalcemia may require urgent cardiac surveillance in addition to metabolic management. Similarly, the progressive bilateral lower limb weakness and altered responsiveness initially raised suspicion for Guillain-Barré syndrome. Hypercalcemia may impair neuromuscular function, resulting in hypotonia, weakness, and altered consciousness [3]. In this context, normal EMG and spinal MRI findings made Guillain-Barré syndrome less likely, while the broader metabolic and hematological workup redirected the diagnosis. This diagnostic pitfall underscores the importance of measuring serum calcium in children presenting with unexplained progressive neuromuscular deterioration. 

Suppressed PTH levels were a key biochemical finding, arguing against primary hyperparathyroidism and orienting the workup toward PTH-independent causes. The combination of elevated LDH and uric acid, aregenerative microcytic anemia, and a normal peripheral blood count without circulating blasts raised a strong suspicion for an underlying hematological malignancy. 

Skeletal radiography was the diagnostic turning point in this case, as reported in several previously published cases (Table 2). The identification of bilateral osteolytic lesions with metaphyseal radiolucent bands on plain radiographs of the lower limbs directly prompted bone marrow aspiration. Whole-body CT scan further revealed more extensive skeletal involvement than anticipated from plain radiography, including diffuse axial disease with cortical disruption and multilevel vertebral compression. Skeletal imaging should therefore be considered early in the workup of any child with severe unexplained hypercalcemia, particularly when associated with neuromuscular symptoms, elevated cell-turnover markers, or unexplained anemia.

The precise mechanism of hypercalcemia in this case remains undetermined, as PTHrP was not measured due to technical limitations. Two principal pathways have been proposed in pediatric ALL. The first involves ectopic PTHrP secretion by leukemic blasts, which may activate the RANK/RANKL/OPG axis, stimulate osteoclast-mediated bone resorption, and enhance renal calcium reabsorption [7,14]. The second, PTHrP-independent pathway involves pro-inflammatory cytokines such as TNF-alpha, IL-1, and IL-6, which can promote osteoclast differentiation and bone resorption [8]. As shown in Table 2, PTHrP was not measured in several published pediatric cases, reflecting practical limitations in routine clinical settings. The absence of t(17;19)/E2A-HLF in our patient, a translocation reported in association with PTHrP-mediated hypercalcemia [7], suggests that alternative mechanisms may have contributed, although this cannot be confirmed without direct PTHrP measurement.

The progressive recovery of PTH levels (4 → 7 → 26 pg/mL) alongside normalization of serum calcium and phosphorus is consistent with reversal of PTH suppression after correction of severe hypercalcemia. However, because calcium normalization occurred before chemotherapy initiation, this recovery should not be interpreted as evidence of leukemic clone eradication. It more likely reflects the effect of initial aggressive hydration and subsequent bisphosphonate therapy on calcium homeostasis.

Regarding management, aggressive intravenous hydration at 3 L/m²/day, initiated with isotonic saline (0.9% NaCl) followed by a glucose-based solution, was used to promote renal calcium excretion. Glucocorticoids were appropriately withheld pending diagnostic confirmation, as premature use can alter blast morphology and delay diagnosis [10]. Zoledronic acid (0.03 mg/kg per dose, four doses total) was used as a temporizing measure to reduce osteoclast-mediated bone resorption [11,12]. Serum calcium progressively normalized before chemotherapy initiation after initial aggressive hydration followed by repeated zoledronic acid administration.

This favorable biochemical evolution supports the usefulness of early calcium-lowering therapy, although the relative contribution of each therapeutic step cannot be determined from a single case. As shown in Table 2, several published cases required chemotherapy initiation to achieve calcium normalization [2,5], whereas others normalized under bisphosphonates combined with hydration [3]. Zoledronic acid is not formally approved for routine pediatric use, but case reports have documented its use at doses ranging from 0.025 to 0.05 mg/kg [11,12]. Clinicians should monitor for hypocalcemia, hypophosphatemia, and acute-phase reactions following administration.

Severe PTH-independent hypercalcemia associated with osteolytic lesions and unexplained neuromuscular symptoms should prompt early hematological investigation, even in the absence of circulating blasts or organomegaly. The diagnostic delay observed in this case, spanning one month across three clinical settings, underscores the value of early skeletal imaging as an accessible and pivotal diagnostic tool.

## Conclusions

We reported a rare and diagnostically challenging case of severe PTH-independent hypercalcemia revealing precursor B-cell ALL in a two-year-old male. The diagnostic course across three clinical settings, the ECG finding of QT interval shortening, the absence of circulating blasts or palpable organomegaly, and the pivotal role of skeletal radiography in guiding bone marrow aspiration all contributed to a complex but ultimately accurate diagnosis. Serum calcium normalized before chemotherapy initiation after initial aggressive intravenous hyperhydration, followed by repeated zoledronic acid administration, while progressive PTH recovery supported reversible PTH suppression after the correction of severe hypercalcemia. Four key clinical messages emerge from this case: (1) serum calcium should be measured in any child with unexplained progressive neuromuscular symptoms; (2) suppressed PTH in severe hypercalcemia should prompt evaluation for PTH-independent causes, including hematological malignancy, even in the absence of circulating blasts; (3) skeletal radiography is an accessible diagnostic tool that may directly orient clinicians toward bone marrow aspiration when osteolytic lesions or metaphyseal radiolucent bands are present; and (4) early multidisciplinary collaboration is essential to reduce diagnostic delay and optimize outcomes.
